# A cross-sectional study of low HIV testing frequency and high-risk behaviour among men who have sex with men and transgender women in Lima, Peru

**DOI:** 10.1186/s12889-015-1730-5

**Published:** 2015-04-21

**Authors:** Sky W Lee, Robert G Deiss, Eddy R Segura, Jesse L Clark, Jordan E Lake, Kelika A Konda, Thomas J Coates, Carlos F Caceres

**Affiliations:** Division of Infectious Diseases, University of California, Los Angeles, California USA; Division of Global Public Health, University of California, La Jolla, San Diego, California USA; Unidad de Salud Sexualidad y Desarrollo Humano, Universidad Peruana Cayetano Heredia, Lima, Peru

**Keywords:** HIV, Testing, Frequency, Behaviour, MSM, Transgender, Peru

## Abstract

**Background:**

Increased HIV testing frequency among high-risk populations such as men who have sex with men (MSM) and male-to-female transgender women (TW) can lead to earlier treatment and potentially reduce HIV transmission.

**Methods:**

We analyzed baseline survey data from 718 high-risk, young (median age 29 [interquartile range 23–35]) MSM/TW enrolled in a community-based HIV prevention trial between 2008–2009. Participants were recruited from 24 neighborhoods in and around Lima, Peru. We assessed HIV testing frequency, testing behaviour, and motivations and barriers to testing. Multivariate analysis identified correlates to prior HIV testing.

**Results:**

Overall, 79.6% reported HIV testing within their lifetimes, however, only 6.2% reported an average of two tests per year, as per Peruvian Ministry of Health guidelines. The most commonly reported motivators for testing were to check one’s health (23.3%), lack of condom use (19.7%), and availability of free testing (14.0%), while low self-perceived risk for HIV (46.9%), fear of a positive result (42.0%), and lack of access to testing services (35.7%) were the most frequently reported barriers. In multivariate analysis, factors independently associated with HIV testing included age [adjusted prevalence ratio (APR) 1.00, 95% CI (1.00-1.01)], transgender-identification vs. gay-identification [APR 1.11, 95% CI (1.03-1.20)], history of transactional sex [APR 1.16, 95% CI (1.07-1.27)], and prior sexually transmitted infection diagnosis [APR 1.15, 95% CI (1.07-1.24)].

**Conclusions:**

An overwhelming majority of participants did not meet the standard-of-care for testing frequency. The reported motivations and barriers to testing highlight issues of risk perception and accessibility. Our findings suggest utilizing non-traditional outreach methods and promoting HIV testing as a routine part of healthcare in Peru to encourage testing and knowledge of HIV serostatus.

## Background

The complex issue of HIV prevention requires a multidimensional approach that includes regular voluntary HIV testing and counseling (HTC), especially among key populations such as men who have sex with men (MSM) and male-to-female transgender women (TW) [1,2]. Knowledge of HIV serostatus via frequent and routine testing is a critical component of comprehensive HIV prevention that triggers the cascade of care for those who are HIV-positive while providing an important opportunity for risk reduction counseling for those who are HIV-negative [2,3]. Infrequent testing remains an obstacle for maintaining accurate HIV serostatus awareness even among people who have previously been screened [4].

Psychosocial and operational barriers [5-8] prevent people from testing for HIV and knowing their serostatus, at times leading individuals to wait to test until they have progressed to the later stages of HIV [7,9]. Perceived high costs of testing, lack of knowledge of testing sites, limited access to testing services and low self-perceived HIV risk continue to hinder routine HIV testing practices [5,10,11]. Low self-perceived risk for HIV exists despite the fact that condom use in Peru is relatively low, particularly with new partners [12]. In addition, several studies have found that HIV-status is generally not discussed between sex partners [13,14]. A study among Peruvian MSM found 27.4% of HIV-positive participants were unaware of their serostatus, with relatively low condom use reported by both HIV-infected and -uninfected participants [13], emphasizing the need for more frequent testing. Nonetheless, despite the cost-effectiveness of HTC programs for emphasizing the benefits of serostatus awareness [9,15], widespread utilization among high-risk groups in Peru has been insufficient.

The current standard of care recommended by the Peruvian Ministry of Health (MoH) for MSM/TW is HTC every six months [16], however, few MSM/TW adhere to this guideline and/or are aware of it. Improved understanding of HIV testing attitudes and practices among MSM/TW in Peru is important for the development of new interventions to improve the frequency of routine counseling and testing. Thus, the aims of our study were as follows: (1) to identify the history and frequency of HIV testing among low-income MSM/TW in Lima, Peru; (2) to assess the relationship between sexual risk behaviours and HTC; (3) and to identify motivations and barriers to HIV testing.

## Methods

### Study design

We conducted a cross-sectional analysis of baseline survey data from the *Comunidades Positivas* trial [17,18]. This randomized, controlled community-based trial examined a combination of structural interventions to encourage community-building and partner therapy for STIs, in order to reduce the incidence of HIV and promote health-seeking behaviours among MSM/TW in and around Lima, Peru. Community development interventions included the creation of community centers and training peer leaders in HIV education, counseling, and leadership skills. Interventions for enhanced partner treatment included targeted campaigns to encourage health-seeking behaviours and provision of educational materials and patient-delivered partner therapy for treatment of bacterial STIs.

Traditional ethnographic methods were used to identify 24 low-income neighborhoods that had a strong network of MSM/TW. Potential participants were recruited using a snowball technique. Inclusion criteria included biologically born males aged 18 to 45 years who reported at least one sexual encounter with a man or transgender woman in the past 12 months, lived or worked near one of the 24 identified neighborhoods, planned to stay in the area for the duration of the study and willing to participate in the study. Baseline survey data was collected between 2008–2009. Eligible participants provided written informed consent prior to the initiation of any study procedures. The study was approved by the Institutional Review Boards of University of California, Los Angeles and Universidad Peruana Cayetano Heredia.

### Data collection

The baseline survey interview was conducted face-to-face by a native Spanish speaker for all sections except for the HIV testing history, which was administered by audio computer-assisted self-interviewing (ACASI) due to fears of reporting bias among HIV positive participants given stigmatization of people living with HIV. Variables assessed included socio-demographic characteristics, HIV testing history (motivations, barriers, number of tests, timing and result of most recent test), a sexual risk assessment (including detailed questions concerning the three most recent sexual contacts), and a problem drinking assessment using the CAGE instrument [19].

### Laboratory methods

All participants underwent pre-test counseling for HIV and syphilis after which rectal and pharyngeal swab samples and 10 ml of blood were collected for testing. Test results and post-test counseling were provided within two weeks of sample collection. Participants with syphilis were given appropriate antibiotic therapy. Participants with newly diagnosed HIV infections were referred to designated MoH facilities for ongoing care and treatment. HIV antibody status was determined using Genetic Systems HIV-1/HIV-2 Plus O EIA (BIO-RAD Laboratories, Redmond, WA) and positive results were confirmed with GenScreen HIV-1/HIV-2 Western Blot (BIO-RAD Laboratories, Redmond, WA). Syphilis infection was determined by Rapid Plasma Reagin (RPR; RPR-nosticon II, BioMerieux, Boxtel, Netherlands), followed by TPPA confirmation (Fujirebio, Japan) and serial dilution of RPR titers. For the purposes of this analysis, diagnosis of syphilis infection was based on an RPR titer of 1:8 or higher. For quality control, 10% of HIV samples were sent to the San Francisco Department of Public Health laboratory (San Francisco, CA), and 10% of syphilis samples were sent to the US Navy Medical Research Unit-6 Bacteriology laboratory in Lima, Peru for confirmatory testing.

### Data analysis

The main outcome of “HIV testing” was based on participant self-report and defined as a dichotomous variable according to lifetime testing history (ever-testers and never-testers). “HIV testing” was also assessed as a continuous variable (according to the number of reported lifetime HIV tests) in order to determine testing frequency. In the descriptive analysis, median values and interquartile ranges (IQR) were estimated for continuous variables and proportions calculated for categorical variables. “Ever-testers” were defined as participants who reported undergoing HIV testing prior to the baseline survey and “never-testers” were defined as those who reported no prior history of HIV testing. Participants were defined as newly HIV-positive if they reported HIV-negative status but had a positive Western blot test at baseline. Participants who accurately reported their HIV-positive status at baseline were considered to have a known HIV-infection, and these individuals were excluded from further analysis.

Survey questions on testing motivations and barriers permitted multiple answers. The possible answer choices were provided from a previously defined list of possible responses with a write-in answer space available. The write-in answers that fell underneath the scope of the pre-defined list of answers were included in the analysis. Write-in answers that fell outside of the scope of pre-defined answers were minimal and not included in the analysis. The mean number of tests per sexually active year was calculated by dividing the number of prior HIV tests by the number of sexually active years since age 18. Self-reported sexual behaviours with the previous three partners were pooled to identify whether individuals had engaged in unprotected anal intercourse (UAI) within the past six months. Problem drinking was defined as answering “yes” to two or more of the four CAGE questions [19].

We used chi-square and Wilcoxon rank sum tests to evaluate associations between the outcome and categorical and continuous variables respectively. To measure the strength of association between the main outcome and independent variables, prevalence ratios (PR) with 95% confidence intervals (CI) were calculated for bivariate and multivariate analyses. We used Poisson-family generalized linear models with a logarithmic link function and robust variance to estimate PR for correlates of ever HIV testing [20,21]. Variables that achieved statistical significance (p < 0.05) in the chi-square or Wilcoxon rank sum tests were included in the bivariate analysis and variables that achieved statistical significance with the outcome of HIV testing in the bivariate analysis were included in the multivariate analysis. Missing data was minimal and excluded from the analysis. For all statistical analyses we used STATA 12.0 (Statacorp, College Station, TX 2011).

## Results

### Study population

Complete clinical and demographic characteristics of the study sample are shown in Table 1.

**Table 1:** **Socio-demographic factors and risk behaviours of ever-testers and never-testers**

**Characteristic**		**Overall**	**Ever-testers (%)**	**Never-testers (%)**	**P-value**	**Prevalence ratio (95% CI)**	^**Ŧ**^ **Adjusted prevalence ratio (95% CI)**
Total		718	559 (79.6)	143 (20.4)	-	-	-
Age (years)							
	Median (interquartile range)	29 (23–35)	29 (24–36)	26 (21–32)	**<0.01**	**1.01 (1.004-1.020)**	**1.00 (1.003-1.010)**
Marital status							
	Married/Cohabitating	26 (3.62)	21 (3.76)	5 (3.50)	0.78	─	─
	Divorced/Separated	15 (2.09)	13 (2.33)	2 (1.40)		─	─
	Single	677 (94.3)	525 (93.9)	136 (95.1)		─	─
Sexual identity							
	Gay/Homosexual	465 (64.8)	345 (61.7)	110 (76.9)	**<0.01**	ref	─
	Transgender	208 (29.0)	180 (32.2)	22 (15.4)		**1.18 (1.09-1.26)**	**1.11 (1.03-1.20)**
	Heterosexual/Bisexual	45 (6.27)	34 (6.08)	11 (7.69)		1.00 (0.84-1.19)	1.00 (0.85-1.18)
Education							
	≤ High School	514 (71.6)	392 (70.1)	108 (75.5)	0.20	ref	─
	> High School	204 (28.4)	167 (29.9)	35 (24.5)		1.05 (0.98-1.14)	─
Prior STI diagnosis by a healthcare worker							
	Yes	221 (30.8)	195 (34.9)	21 (14.7)	**<0.01**	**1.23 (1.13-1.33)**	**1.15 (1.07-1.24)**
	No	497 (69.2)	364 (65.1)	122 (85.3)		ref	─
Number of sexual partners in the past 6 months							
	Median (interquartile range)	4 (2–15)	5 (2–20)	3 (2–7)	**<0.01**	0.99 (0.99-1.00)	─
Newly HIV-positive							
	Yes	82 (11.4)	61 (10.9)	18 (12.3)	0.57	─	─
	No	636 (88.6)	498 (89.1)	125 (87.4)		─	─
Positive RPR							
	Yes	170 (23.7)	144 (26.0)	24 (17.1)	**<0.05**	**1.10 (1.02-1.19)**	0.99 (0.91-1.07)
	No	548 (76.3)	410 (74.0)	116 (82.9)		ref	─
Problem drinking							
	Yes	415 (57.8)	334 (59.8)	70 (49.0)	**<0.05**	**1.09 (1.01-1.18)**	1.05 (0.97-1.13)
	No	303 (42.4)	225 (40.3)	73 (51.1)		ref	─
Sexual role in prior 6 months							
	Receptive	440 (61.3)	341 (61.2)	88 (62.0)	0.44	─	─
	Insertive	214 (29.8)	173 (31.1)	38 (26.8)		─	─
	Both	31 (4.32)	21 (3.77)	9 (6.34)		─	─
	Neither	30 (4.18)	22 (3.95)	7 (4.93)		─	─
Transactional sex							
	Yes	394 (54.9)	332 (59.4)	50 (35.0)	**<0.01**	**1.23 (1.13-1.33)**	**1.16 (1.07-1.27)**
	No	324 (45.1)	226 (40.4)	93 (65.0)		─	─
Engaged in unprotected intercourse in past 6 months							
	Yes	443 (61.7)	347 (62.1)	86 (60.1)	0.67	1.02 (0.94-1.10)	─
	No	275 (38.3)	212 (37.9)	57 (39.9)		ref	─
Engaged in unprotected insertive anal intercourse in past 6 months							
	Yes	131 (18.2)	97 (17.4)	32 (22.4)	0.17	0.93 (0.84-1.04)	─
	No	587 (81.8)	462 (82.7)	111 (77.6)		ref	─
Engaged in unprotected receptive anal intercourse in past 6 months							
	Yes	469 (65.3)	369 (66.0)	87 (60.8)	0.25	1.05 (0.97-1.14)	─
	No	249 (34.7)	190 (34.0)	56 (39.2)		ref	─

^Ŧ^Adjusted for age, sexual identity, prior STI diagnosis by a healthcare worker, positive RPR, problem drinking and transactional sex. Numbers in bold have p-value <0.05.

The sample consisted of 718 participants with a median age of 29 (IQR 23–35) years and a median number of sexually-active years of 14 (IQR 9–21). Participants identified most commonly as gay (64.8%) or transgender (29.0%), and most had not obtained post-secondary education (71.6%). Many participants reported high-risk behaviours such as transactional sex, (which was defined as trading sexual activities for money or goods; 54.9%), UAI within the past six months (61.7%), or problem drinking, as denoted by a positive CAGE screen (57.8%).

### HIV testing practices

Of the 718 participants, the majority (79.6%) reported at least one HIV test within their lifetime (Table 1). Among all participants, the average testing frequency was 0.54 (Standard Deviation 0.85) tests per sexually active year since age 18. Only 6.2% of participants reported an average of two tests per year since the age of 18, as per MoH guidelines. Ever-testers had a median of three (IQR 2–6) lifetime tests, and less than half (42.3%) had tested within the past year. Previous testing did not guarantee serostatus awareness, as 72 (12.9%) ever-testers reported not returning for their test results. Among this group, the most common reason for not returning for their results was “I didn’t have time” (54.2%).

A complete list of reported motivations and barriers is shown in Table 2. The most commonly reported motivation for HIV testing among ever-testers was to “check health” (50.4%). Only 14.8% of ever-testers reported receiving a recommendation to test for HIV by a healthcare professional, even though almost 35.0% reported a prior STI diagnosis. The most common testing barrier among never-testers was low self-perceived risk for HIV infection (46.9%).

**Table 2:** **Motivations and barriers to HIV testing**

**Motivation**	Ever-testers N = 559 (%)*	**Barrier**	Never-testers N = 143 (%)*
To check my health	282 (50.4)	I do not believe I am at risk	67 (46.9)
I had sex without a condom	240 (42.9)	I am afraid to receive the result	60 (42.0)
I was offered a free test	170 (30.4)	I have not had the opportunity	51 (35.7)
I wanted to know if I was HIV positive	165 (29.5)	I am afraid other people will find out my result	32 (22.4)
One of my partners did not use a condom	92 (16.5)	I am afraid of needles	31 (21.7)
It was recommended by a healthcare professional	83 (14.8)	I do not know where to get tested	18 (12.6)
It was required for a procedure/work/visa/marriage/etc.	52 (9.30)	I am afraid of my partner’s reaction	17 (11.9)
For another research study	48 (8.59)	Other people will think that I am sick	15 (10.5)
One of my partners wanted me to get tested	38 (6.80)	I do not have money to pay for the test	14 (9.79)
I had AIDS symptoms	20 (3.58)	Other	8 (5.59)
One of my partners had AIDS symptoms	15 (2.68)	No response	5 (3.50)
Other	8 (1.43)		
I don’t know	3 (0.54)		

*Percentages were calculated from total number of ever- testers or never-testers not by total number of responses.

Overall, 82 participants (11.4%) were newly diagnosed with HIV of whom 30 (36.6%) tested within the past year, 31 (37.8%) tested over a year ago, and 18 (22.0%) had never been tested. Three participants with newly diagnosed HIV infection (3.66%) declined to answer whether they had ever been tested.

### Testing and behaviour

Although the HIV prevalence between ever-testers and never-testers was similar (10.9% vs. 12.3%, p = 0.57), ever-testers were significantly more likely to engage in transactional sex, report problem drinking, have a positive RPR, and report greater number of sexual partners in the past six months than never-testers (Table 1). In the multivariate analysis, factors independently associated with ever having received an HIV test included older age [Adjusted Prevalence Ratio, APR, 1.00, 95% CI (1.00-1.01)], identification as transgender as compared with gay [APR 1.11, 95% CI (1.03-1.20)], engaging in transactional sex [APR 1.16, 95% CI (1.07-1.27)], and receiving a prior STI diagnosis by a healthcare worker [APR 1.15, 95% CI (1.07-1.24)].

## Discussion

In our study, 94% of participants did not meet the Peruvian MoH standard for MSM/TW of testing for HIV every 3–6 months [16] and approximately 60% of the participants who were newly diagnosed with HIV infection had not been tested in the previous year. The reported barriers and motivations for HTC emphasize issues of risk perception and accessibility and identified areas for improvement. Interestingly, ever-testers were significantly more likely to engage in a number of high-risk behaviours than never-testers, including transactional sex. While this study was conducted between 2008–2009, Peruvian MOH guidelines continue to emphasize traditional HTC programs among vulnerable populations, and our findings suggest a need for innovative outreach methods for MSM/TW who lack access to HIV testing.

Our finding of high prevalence of risk behaviour among ever-testers is not uncommon [22,23] but nonetheless denotes an interesting trend. A hallmark of HTC programs is the provision of pre- and post-test counseling, which is offered as a means of risk reduction for individuals who test negative for HIV. Our results, however, demonstrate the shortcomings of this approach, as having been tested did not result in any apparent modification of risk behaviours among our participants. In fact, the opposite trend was observed. Indeed, some individuals may have sought false reassurance through regular HIV testing while continuing to engage in a number of risk behaviours [4]. To address the first issue, the World Health Organization now emphasises the importance of rapid testing, which would facilitate learning one’s serostatus and possibly promote behaviour change on a more immediate basis [3], though rapid HIV testing has not been widely implemented in Peru.

Our finding that transactional sex and prior STIs diagnoses were independently associated with a history of HIV testing provides further evidence that individuals engaging in high-risk practices recognized the need for HIV testing or were offered tests after STI diagnosis. Nonetheless, fewer than half of individuals who reported a prior STI reported receiving a referral for HIV testing by their healthcare provider. It is likely that these individuals were a part of the demographic heavily targeted by the MoH periodic medical check-ups for key populations to increase HIV testing rates [10]. Our data suggests that present strategies are working to get individuals most at risk to test at least once, but testing frequency can be improved, and an opt-out model for HIV testing at the time of STI screening, as has been successfully implemented in other low-resource settings, may provide added benefit [24]. Newer innovations which utilize non-traditional approaches to HIV testing, including social media and mobile testing sites, also offer promise [25,26]. As an example, Project Accept - a large multi-country, community-based randomized control trial to promote community-based HTC – showed that with community involvement, HTC in mobile units can increase the frequency of repeat testing [15].

Motivations and barriers to HIV testing in our study included issues of risk perception and accessibility, providing some insight into the low frequency of prior HIV testing and identifying areas for improvement among MSM/TW in Peru. Until 2004, when antiretroviral treatment became widely available, there were few incentives for routine testing [27], which may explain low testing frequency over our participants’ lifetimes, but this does not explain why the majority of ever-testers did not test within the past year. For those without access to routine healthcare, some practical steps to facilitate HIV testing include making available free or low-cost testing locations and providing testing outside of traditional healthcare facilities such as in saunas or clubs instead of STI clinics. Decreasing the stigma associated with HIV testing, by promoting it as a more common and routine test, may also increase testing frequency among groups at highest risk of infection [11]. Similarly, as HIV care is increasingly recognized as a manageable chronic disease and stigma is reduced, further barriers to testing may be overcome, such as the fear of a positive result or the reactions of others to a positive HIV test. In sum, the reported motivations and barriers illustrate the importance of establishing social norms for routine testing among MSM/TW, utilizing the current MoH program strategies and creating alternative outreach methods for MSM/TW not reached otherwise.

Our study had several limitations. As a cross-sectional, secondary data analysis, it was not specifically designed to address issues surrounding HIV testing and cannot draw causal inferences with regard to risk behaviours and testing among MSM/TW. However, the high proportion of new HIV diagnoses and reported risk behaviours indicate missed opportunities for behaviour change. The sensitive nature of some of the questions may have caused some participants to misrepresent themselves, although all attempts were made to maintain privacy and confidentiality. Data on motivations and barriers for prior testing were limited by a pre-defined set of options, as well as write-in categories, and may not have captured all the factors influencing HIV testing (e.g. transphobia uniquely affecting TW), as the questions did not differentiate between primary and secondary factors. Nonetheless, the majority of write-in categories were reiterations of the potential responses provided, which is consistent with prior studies conducted within and outside of Peru [5,6,8].

A final note is warranted with respect to our study population. In Peru, the social networks of MSM and TW tend to overlap, and it is a frequent challenge of studies to characterize the distinct attributes of each group. Moreover, at the time of study design, these group differences were not as well recognized as they are today, and the development of improved study methodologies continues to be necessary for an improved understanding of sexual dynamics among Peruvian MSM/TW. Nonetheless, our sample population involved high-risk, low-income MSM/TW, and while not representative of all MSM/TW in Lima, Peru, it highly corresponded with those targeted by MoH HIV testing programs.

## Conclusions

Our study calls for alternative outreach methods and public education to improve adherence to the MoH recommended standard of care for HIV testing among MSM/TW through a norm of client-initiated HTC. The use of mobile testing and availability of HIV testing in MSM/TW frequented venues would capture MSM/TW not reached by traditional healthcare approaches, which places responsibility on patient-initiated visits to an STI clinic [28,25]. In addition to operational barriers, an emphasis on education to change the perception of HIV testing as a routine part of healthcare may increase adherence to the standard of care. Future studies addressing attitudes regarding HTC and alternative testing venues and methods would provide practical recommendations to facilitate routine testing for key populations such as MSM/TW in Peru.

## Abbreviations

ACASI: Audio computer-assisted self-interviewing
APR: Adjusted prevalence ratio
CI: Confidence interval
HTC: HIV testing and counseling
IQR: Interquartile range
MOH: Ministry of health
MSM: Men who have sex with men
STI: Sexually transmitted infection
TW: Transgender women
UAI: Unprotected anal intercourse
